# Nanomaterials in Immunology: Bridging Innovative Approaches in Immune Modulation, Diagnostics, and Therapy

**DOI:** 10.3390/jfb15080225

**Published:** 2024-08-14

**Authors:** George-Alexandru Croitoru, Diana-Cristina Pîrvulescu, Adelina-Gabriela Niculescu, Dragoș Epistatu, Marius Rădulescu, Alexandru Mihai Grumezescu, Carmen-Larisa Nicolae

**Affiliations:** 1 Faculty of Dental Medicine, Carol Davila University of Medicine and Pharmacy, 8 Eroii Sanitari Street, 050474 Bucharest, Romania; alex.croitoru@umfcd.ro (G.-A.C.); dragos.epistatu@umfcd.ro (D.E.); carmen-larisa.nicolae@umfcd.ro (C.-L.N.); 2 Faculty of Chemical Engineering and Biotechnology, National University of Science and Technology Politehnica Bucharest, 011061 Bucharest, Romania; diana.pirvulescu@stud.fim.upb.ro (D.-C.P.); marius.radulescu@upb.ro (M.R.); agrumezescu@upb.ro (A.M.G.); 3 Research Institute of the University of Bucharest—ICUB, University of Bucharest, 050657 Bucharest, Romania

**Keywords:** nanotechnology, nanoparticles, immunology, immune response, vaccine development, immunotherapy, nanomaterials

## Abstract

The intersection of immunology and nanotechnology has provided significant advancements in biomedical research and clinical applications over the years. Immunology aims to understand the immune system’s defense mechanisms against pathogens. Nanotechnology has demonstrated its potential to manipulate immune responses, as nanomaterials’ properties can be modified for the desired application. Research has shown that nanomaterials can be applied in diagnostics, therapy, and vaccine development. In diagnostics, nanomaterials can be used for biosensor development, accurately detecting biomarkers even at very low concentrations. Therapeutically, nanomaterials can act as efficient carriers for delivering drugs, antigens, or genetic material directly to targeted cells or tissues. This targeted delivery improves therapeutic efficacy and reduces the adverse effects on healthy cells and tissues. In vaccine development, nanoparticles can improve vaccine durability and extend immune responses by effectively delivering adjuvants and antigens to immune cells. Despite these advancements, challenges regarding the safety, biocompatibility, and scalability of nanomaterials for clinical applications are still present. This review will cover the fundamental interactions between nanomaterials and the immune system, their potential applications in immunology, and their safety and biocompatibility concerns.

## 1. Introduction

**Figure 1:**
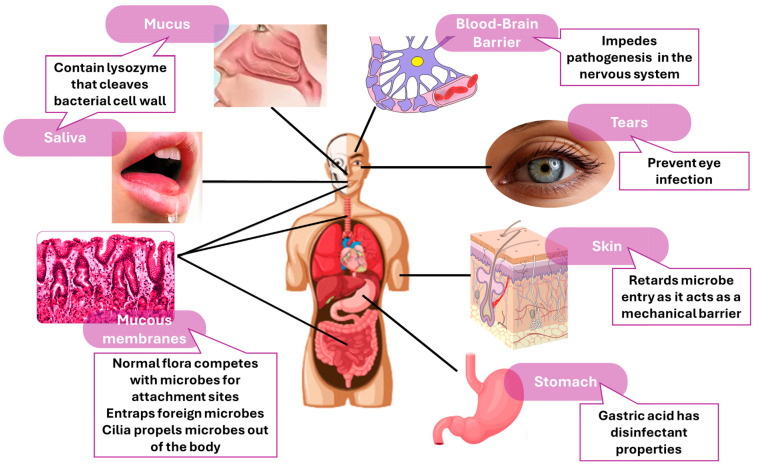
Schematic illustration of innate immunity barriers. Created based on information from [1,6,7,8].

Immunology is the science that studies all the functions of the immune system, or immunity. The immune system is the body’s defense mechanism against harmful microorganisms (like bacteria, fungi, and viruses) and protects it against infections and diseases. The immune system consists of a complex network of cells, tissues, and organs, such as bone marrow, skin, spleen, and mucous membranes, and it recognizes and neutralizes the intruders while protecting the healthy cells and organs. It is divided into innate immunity and adaptive immunity, both of which have roles in sustaining the body’s health and protecting it against disease. Innate immunity provides the first defense mechanism, and it consists of four protective barriers: (a) physical barriers, such as skin and mucous membranes; (b) physiologic barriers, which involve temperature and chemical mediators; (c) endocytic/phagocytic, where specific cells uptake the foreign microorganism; (d) inflammatory, involving the recruitment of different cells (such as macrophages). A schematic illustration of innate immunity’s barriers is presented in Figure 1. Adaptive immunity is the second defense mechanism, and it involves specialized cells, more precisely lymphocytes, that remember and respond more effectively to previously encountered pathogens. Adaptive immunity can be slower than the innate one, but after it fights off one type of pathogen, it will be eradicated much faster the next time it is encountered. The adaptive immunity system is made of B cells and T cells, two distinct groups of lymphocytes and antibodies, and all are responsible for the immune responses [1,2,3,4,5].

There has been a growing interest in combining immunology with nanotechnology in the past few years. This multidisciplinary area of study focuses on creating materials at the nanoscale to control and understand immunological reactions. A few challenges and aims currently exist in immunology, including developing new diagnostic methods and treatments for immune diseases and improving immune responses to vaccines and infections. These shortcomings can be addressed by using nanotechnology [9,10]. Specifically, overcoming these issues involves using nanomaterials, materials between 1 and 100 nm with unique properties such as small size, shape, and structure, which can be modified to fit the desired use. These materials can be designed to modulate immune functions by interacting with immune cells. Nanomaterials could be applied in the field of immunology by being used as carriers for drugs, antigens, or nucleic acids [10,11].

Researchers aim to improve medical procedures, like diagnostics and treatments in immunology, with the help of nanomaterials. Because of their small size and high area-to-volume ratio, therapeutic drugs can be loaded more efficiently and in higher quantities, resulting in effective delivery to specific cells or tissues. This leads to an increased therapeutic effect and reduced side effects because only diseased cells are targeted without affecting healthy ones [11,12]. Nanomaterials have promising effects that can make them suitable for diagnostics. Researchers aim to create extremely specific and sensitive biosensors that can identify biomarkers at low concentrations, making early diagnosis possible, which could improve patient outcomes in future applications. For instance, pathogens or proteins linked to disease can be precisely captured and detected using nanoparticles (NPs) linked with antibodies or other ligands [13,14].

Nanomaterials have mainly demonstrated their beneficial effects in treating autoimmune diseases and cancer, among other immune-related conditions. Immunomodulatory drugs can be directly delivered to immune cells via NPs, changing their activity and improving the body’s natural immunological response. In cancer therapy, nanomaterials can be used to deliver chemotherapeutic drugs directly to tumors, so the damage to healthy cells is reduced and the therapeutic effect is improved [15,16].

Nanomaterials can cross biological barriers like the blood–brain barrier, thereby creating alternative treatments for neurological diseases. Furthermore, nanomaterials show promise in more durable vaccine development because they may trigger prolonged immune responses [16,17].

The application of nanotechnology to immunology can open new avenues for research. In this way, it will be possible to gain a deeper understanding of how immune mechanisms work and how they can be manipulated to improve the immune response. As nanotechnology research advances, it has the potential to improve nanomedicine by providing great alternatives for the detection and treatment of a variety of diseases. Therefore, this review aims to provide a comprehensive overview of the current state of nanotechnology in immunology, highlighting the possible applications of nanomaterials in immune modulation, diagnostics, and therapy.

## 2. Fundamental Interactions between Nanomaterials and the Immune System

**Figure 2:**
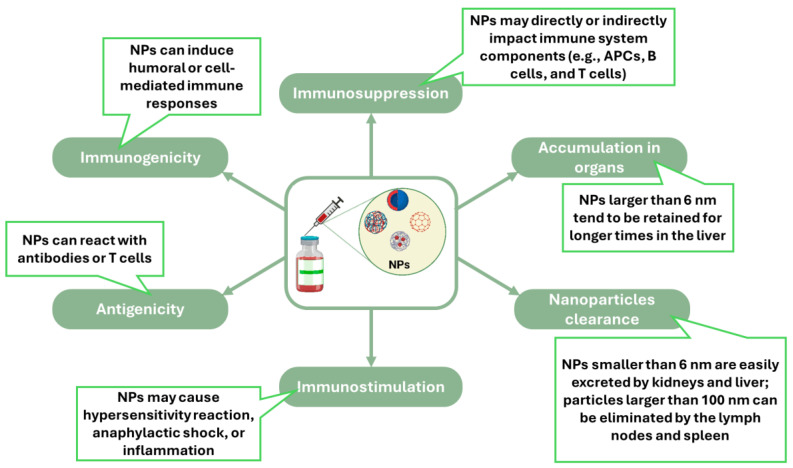
Illustration of how nanomaterials (NPs) can affect the immune system. Created based on information from [18,19,20].

The immune system and nanomaterials can interact in many complex ways that involve different immune cells, signaling pathways, and cellular mechanisms. A schematic illustration of how nanomaterials influence the immune system is represented in Figure 2. Understanding these interactions is crucial so we can fully explore nanomaterials in immunological applications [18].

Phagocytosis is a biological process that allows phagocytic cells (such as macrophages) to engulf foreign cells or materials. These cells are important components of the innate immune system. In some cases, the immune system can recognize nanomaterials as intruders, so they will be engulfed by the phagocytic cells. Numerous immunological reactions, including inflammatory ones and the activation of adaptive immunity, can be triggered by this uptake [18].

**Figure 3:**
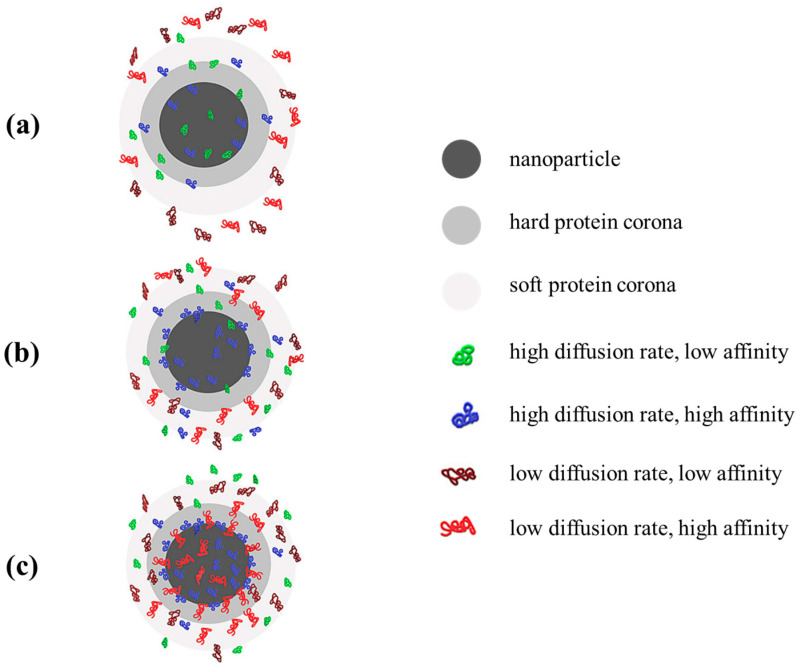
Stages in protein corona development on the nanoparticle surface. (**a**) Initiation of protein corona formation (occurs in the first seconds after the nanoparticle reaches the biological fluid), (**b**) beginning of exchange of proteins with low affinity with proteins that have a higher affinity (within seconds to minutes), and (**c**) stabilized protein corona containing proteins with high affinity in the first layer (hard protein corona) and low-affinity proteins in the second layer (soft protein corona). Adapted from open-access sources [23,24].

The formation of protein corona represents an essential aspect of the interaction between nanomaterials and the immune system (Figure 3). Proteins stick onto the surface of NPs when they come into contact with biological fluids, creating this corona, which has a major impact on how immune cells react to the nanomaterials. The dynamic composition and structure of the protein corona may change over time, and thereby, it affects how the NPs interact with the immune system and how they are perceived. This protein corona alteration can have two different effects. In the first case, the alterations may cause proteins to reveal hidden binding sites that produce an immune response, or they may degrade protein activity. In the second case, proteins’ surface conformational changes can trigger the appropriate cell signaling to aid in medical treatments. To minimize unwanted immune responses and create nanomaterials, it is important to fully understand and regulate the protein corona [21,22].

One technique used to modify nanomaterials’ interactions with the immune system is surface modification. Through the functionalization of the surface with certain ligands, such as peptides, researchers can lead nanomaterials toward particular immune cells or receptors. For instance, adding polyethylene glycol (PEG) coating to NPs can delay the immune system’s detection and removal of the NPs, allowing them to remain in the circulatory system for longer [25,26].

NPs can influence immune responses by affecting how cytokines behave and signaling molecules that facilitate immune cell communication. NPs can either promote or inhibit the production of cytokines, depending on their specific properties. For example, certain NPs can increase the synthesis of proinflammatory cytokines, stimulating an immune response that is beneficial for cancer therapy or vaccine adjuvants. On the other hand, some NPs can inhibit the release of inflammatory cytokines, which may be useful in treating autoimmune diseases or inflammation [27].

The role of nanomaterials as modulators of the immune system has recently become a new area of research. NPs can influence the immune system through different mechanisms. Antigen transport to antigen-presenting cells (APCs), including dendritic cells (DCs), is one of the primary mechanisms. Antigens can be encapsulated in NPs and delivered directly to APCs, increasing T cell activation and the resulting adaptive immunological response. This strategy is very helpful in producing vaccines since NPs can transport antigens and effectively and precisely deliver them to the immune system [28,29,30].

NPs can have an adjuvant function and influence the immune system. These adjuvants are substances that improve the immune system’s reaction to an antigen. NPs can act as adjuvants by creating a retention effect and then gradually releasing the antigen, which results in an extended immune response. Furthermore, pathogen-associated molecular patterns (PAMPs), identified by immune cell pattern recognition receptors (PRRs), can be added to their surface. The innate immune responses triggered by this recognition contribute to the increase in the antigen-specific immune response [31,32,33].

Additionally, immunomodulatory substances or medications can be specifically delivered to the desired immune cells through NPs. With this targeted delivery, immune cell activity can be modulated to either increase or decrease function as desired [34]. For example, to treat autoimmune diseases by lowering inflammation and limiting immune cell overactivation, NPs loaded with immunosuppressive medications can be directed toward T cells or macrophages. On the other hand, immunostimulatory chemicals found in NPs can increase immune cell activity, increasing the body’s defense against cancer [35,36,37]. NPs can also cross biological barriers, like the blood–brain barrier, a highly selective membrane that protects the brain from harmful substances [38]. For instance, by delivering therapeutic compounds directly to the central nervous system, NPs can provide alternatives for treating neuroinflammatory illnesses like Alzheimer’s and multiple sclerosis. By crossing such barriers, NPs allow for localized immune regulation and targeted delivery, lowering systemic negative effects and enhancing the treatment [39,40].

The development of nanotechnology in immunology depends on the relationships between nanomaterials and the immune system. To create NPs that can successfully regulate immune responses, it is first mandatory to fully understand how the immune system reacts to nanomaterials. In addition, by understanding these aspects, researchers can use nanomaterials in diverse biomedical applications, which will be discussed in the next sections. Moreover, safety and ethical issues must be considered when developing NPs for immunological purposes. An important factor to consider is the possible toxicity of NPs because they can trigger inflammation and oxidative stress. In addition, the long-term effects of their exposure on the immune system are not known yet [41].

**Figure 4:**
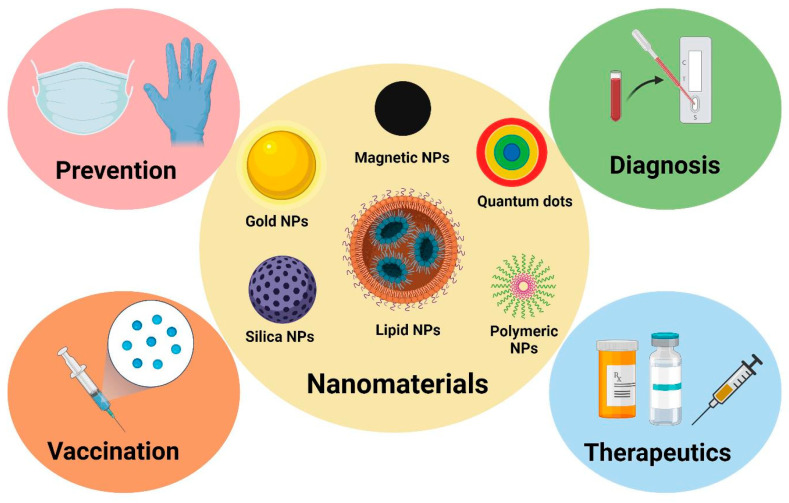
Nanomaterials’ potential applications in immunology. Reprinted from an open-access source [42].

Nanomaterials have many applications in immunology, as presented in Figure 4, which will be further discussed.

## 3. Nanomaterials in Immunodiagnosis

Conventional diagnostic methods can encounter challenges in detecting low concentrations of biomarkers, which are important indicators of disease presence or progression [41]. Due to their properties, nanomaterials can address these challenges since they can improve the sensitivity and specificity of immunological diagnostics. Another important application of the NPs is using them as biosensors, thereby offering improved detection with a more rapid, sensitive, and selective effect. It is possible to make nanomaterials that interact with particular biomolecules with great selectivity, including proteins, nucleic acids, and other molecules [43].

For example, gold nanoparticles (AuNPs) can be functionalized to detect certain viruses, as demonstrated in a study by Peng et al. [44]. The researchers developed a highly sensitive method for detecting the SARS-CoV-2 N protein using large AuNPs with a diameter of 150 nm on a surface plasmon resonance (SPR) sensor. These large AuNPs greatly improved detection sensitivity, achieving a sensitivity level ten times higher than that of smaller NPs, which are typically used. Large AuNPs improve SPR sensitivity to femtomolar (fM) levels, which are essential for detecting low concentrations of the SARS-CoV-2 N protein. The detection limit was determined to be 85 fM, which is the highest sensitivity reported for SARS-CoV-2 N protein detection. In another study, Choosang et al. [45] developed a highly sensitive and selective electrochemical immunosensor to measure prostate-specific antigen (PSA). The sensor used a composite material (chitosan, graphene, ionic liquid, and ferrocene) on a screen-printed carbon electrode (SPCE) and formed a 3D porous cryogel decorated with AuNPs. The biocompatible and porous cryogel increased the surface area for AuNPs, improved electron transfer, and amplified the current signal. The sensor showed excellent analytical performance, with a wide detection range and a very low detection limit. It also demonstrated good stability and selectivity. More studies have used AuNPs for detecting pathogens, and promising results have been shown, as summarized in Table 1.

Magnetic NPs (MNPs) can also be used in biosensor development. MNPs can be functionalized with antibodies or other ligands to isolate pathogens or biomarkers from samples. Once captured, the MNPs can be separated from the sample using an external magnetic field, resulting in highly sensitive detection [51]. In a recent study, MNPs were functionalized with monoclonal antibodies to detect *Salmonella typhimurium.* Three assay formats were compared, and the preincubation one-step sandwich assay produced the highest SPR signal. It was observed that the method could detect lower numbers of *Salmonella* without the need for overnight enrichment. The study concluded that SPR combined with MNPs provides a highly sensitive and specific method for detecting *Salmonella typhimurium*, and the approach could be adapted for other pathogens by using specific antibodies [52]. MNPs can also be used to improve the speed and efficiency of protein detection in diagnostics, as demonstrated by Qiu et al. [53]. Their study introduced a new method called plasmonic photothermal (PPT)-immuno-polymerase chain reaction (iPCR) for protein detection. Using plasmonic MNPs and LED-based devices, PPT-iPCR combines immunological identification with the amplification capacity of PCR. This technique speeds up and uses less energy by reducing the amount of time that PCR requires for thermal cycling. It was found that the PPT-iPCR could detect tumor necrosis factor α (TNF-α) proteins at a very low detection limit and faster than commercially available testing kits. The study demonstrated that PPT-iPCR could be used with existing enzyme-linked immunosorbent assays (ELISA), PCR protocols, and commercially available kits without modification, resulting in faster, less expensive, and more accurate detection devices for biomarkers.

In addition to pathogen detection, NP-based biosensors can be used to monitor immune status. For example, immune cells may be labeled and tracked using quantum dots (QDs), allowing for real-time monitoring of immunological responses [54]. A recent study examined the potential of QDs to enhance the effectiveness of DC vaccines in cancer immunotherapy. QDs were synthesized to have multiple roles, including acting as fluorescent nanoprobes for tracking. The QD-pulsed DC vaccines made real-time vaccine tracking possible while also triggering a strong immune response. This strategy reduced the immunosuppressive tumor environment and increased both innate and adaptive immunity. Moreover, this method showed high antigen-specific T-cell immunity in animal studies by substantially inhibiting local tumor growth and preventing tumor spread [55].

These studies demonstrated nanomaterials could have the ability to improve the sensitivity and specificity of diagnostic techniques, making them more accurate and allowing for faster detection, even at low concentrations. Incorporating nanotechnology into the biosensor development process for immune status monitoring and pathogen detection has made diagnostic tools more accurate, quicker, and less expensive. These developments provide alternative ways for detecting diseases earlier and monitoring immune responses.

## 4. Nanomaterials in Vaccine Development

Even though many very effective vaccines are available, some still have limitations, such as instability, low immunogenicity (the capacity for triggering an immune response), and the requirement for repeated administration. Additionally, there are still certain infectious diseases for which no vaccines are available [42,56]. The application of nanotechnology has resulted in significant progress in vaccine development. Due to their characteristics, nanomaterials can aid in the development of vaccines that are simpler to administer, more stable, and more effective. They are important in the fight against infectious diseases and even cancer because of their capacity to increase immune responses and act as carriers in targeted therapy [57,58]. The potential of nanomaterials to increase immune responses is one of the primary advantages of using them in the production of vaccines. Because NPs may be designed to resemble pathogens in size and shape, the immune system can easily recognize them. They can be loaded with adjuvants, which are compounds that stimulate the immune system, and antigens. Additionally, precise control over the release and distribution of vaccine components can be achieved by using nanomaterials. Researchers can ensure that the vaccine reaches its intended location of action by targeting particular cells or tissues by modifying the surface of NPs with different substances or molecules. Vaccines can be safer and more effective because of this targeted administration, which minimizes possible adverse effects and lowers the required dosage [57].

Lipid nanoparticles (LNPs) have been widely investigated for RNA delivery over the past few years. LNPs have four lipid types: ionizable lipids, cholesterol, phospholipids, and PEG lipids. An essential part of LNPs is an ionizable lipid, which possesses a tertiary amine structure that allows RNA to be encapsulated and transported to the cytoplasm. PEG lipids increase the half-life of LNPs, resulting in a longer presence in the circulatory system. Additionally, cholesterol and phospholipids stabilize LNPs. All these properties make them promising for creating RNA-loaded NPs [59,60]. Studies demonstrated that LPNs could be successfully used in cancer vaccines. Shimosakai et al. [61] created mRNA-loaded LPNs for cancer vaccines to target immune cells in the spleen. The LNPs were optimized with a lipid composition to maximize immune cells’ transfection efficiency in the spleen. The researchers found that the created LPNs transfected DCs, macrophages, and B cells in the spleen after intravenous administration. Strong dose-dependent cytotoxic T lymphocyte (CTL) activity was generated by the LNPs, which was significantly higher than that in local administration. The potential of LNPs for treating cancer was demonstrated in mouse studies by their preventive and therapeutic anticancer activities. Sasaki et al. also studied the potential of LNPs in cancer immunotherapy [62]. Their scope was to optimize the size and composition of mRNA-loaded LNPs to efficiently deliver mRNA to splenic DCs for vaccination. Different lipid compositions and sizes (mRNA-loaded A-11-LNPs and MC3-LNPs RNA-LPX) were examined. The most efficient LNPs for splenic DC absorption and gene expression were those with a size between 200 and 500 nm. The A-11-LNP formulation best achieved strong transgene expression and DC maturation. A tumor model derived from mice also showed significant anticancer effects. Chan et al. [63] developed an LNP system called 113-O12B, which is a disulfide bond-containing ionizable lipidoid, to deliver mRNA vaccines specifically to lymph nodes for cancer treatment. 113-O12B LNPs demonstrated superior specificity over the widely used ALC-0315 (a synthetic lipid) LNPs by delivering mRNA to immune cells in lymph nodes without requiring active targeting ligands. In a mouse model, this targeted administration improved the effect against tumors and increased the CD8+ T cell response. mRNA vaccination showed strong tumor suppression because it contained 113-O12B, particularly when combined with anti-PD-1 treatment. Certain mice showed total tumor eradication and long-term immune memory that prevented metastasis.

LNPs have also been used in other vaccine formulations for conditions besides cancer. A few research examples and their findings are summarized in Table 2.

**Figure 5:**
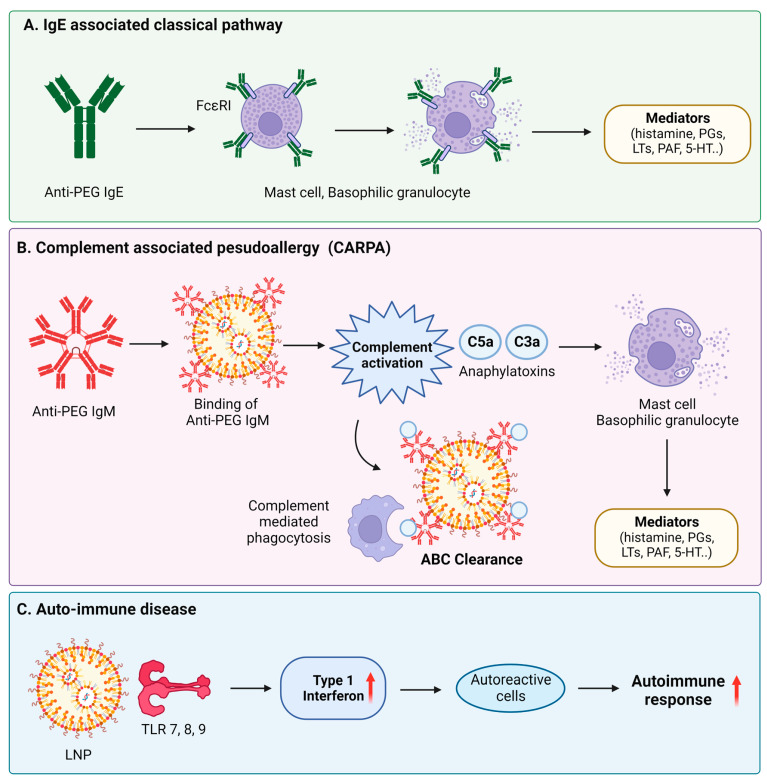
(**A**) Mechanisms of adverse responses caused by LNP-based vaccines. PEGylated lipid nanoparticles (LNPs) induce the production of anti-PEG antibodies in the body, which can result in adverse effects. IgE antibodies attach to FcεRI receptors found on mast cells or basophilic granulocytes, which are crucial cells involved in the acute hypersensitivity response. Several tyrosine kinases activate as mediators. (**B**) When PEGylated liposomes are administered, anti-PEG IgM in the body attaches to the liposomes, causing complement activation through the classical complement pathway. This complex is quickly removed from the blood circulation due to Kupffer cell phagocytosis, known as the accelerated blood clearance phenomenon. This anaphylatoxin triggers inflammatory mediators by activating macrophages, mast cells, and basophils. This mediator binds to receptors of autonomic effector cells, endothelial cells, and smooth muscle cells and stimulates CARPA activation. (**C**) Autoimmunity may appear as a result of mRNA-LNP-based drug administration due to three main reasons: mRNA acts as an autoantigen and induces the autoimmune process through TLR7; the autoimmune process proceeds through the innate immune response to LNPs due to the adjuvant role of these nanoparticles; and the mRNA-LNP vaccine may act toward enhancing the immune process and aggravating the autoimmune response. Reprinted from an open-access source [59].

Despite their great promise and advantageous features, lipid nanoparticles may also cause certain adverse effects. The mechanisms of adverse responses of LNP-based vaccines include IgE-mediated allergy, non-IgE-mediated allergy, and autoimmune reaction [59], as visually represented in Figure 5. Thus, these aspects have to be thoroughly considered when designing novel LNP-based formulations and overcome through surface modification strategies.

**Figure 6:**
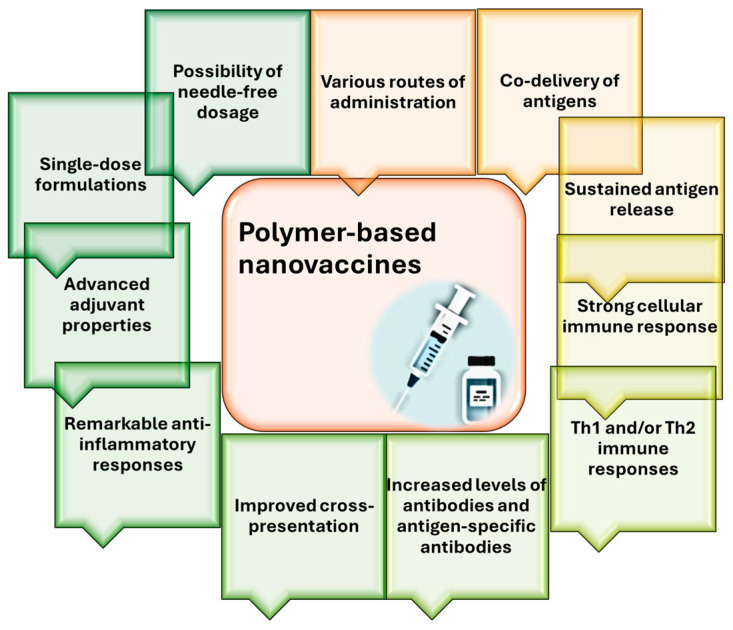
Advantages of utilizing polymeric NPs in vaccine development. Adapted from an open-access source [71].

Polymeric NPs have also been an area of interest in vaccine development (Figure 6). They can encapsulate antigens and protect them from degradation while acting as delivery systems in targeted therapy. These NPs also have natural adjuvant properties that improve immunological responses and allow for the prolonged release of antigens. Polymeric NPs are easily formulated into many types of vaccines because they are biocompatible and biodegradable, reducing toxicity [69,70].

In a recent study, researchers developed a surface-modified poly lactic-co-glycolic acid (PLGA) nanocarrier system to protect a protein-based antigen for potential oral vaccine delivery. The surface-functionalized PLGA NPs demonstrated enhanced protection of the protein antigen in simulated gastric and intestinal fluids compared to non-functionalized NPs. In tests using various coating agents, PLGA-PEG and PLGA-alginate (PLGA-ALG) NPs demonstrated greater stability in simulated intestinal and intestinal fluids than uncoated NPs and PLGA-Eudragit L100 (PLGA-EUD) NPs. Because of their superior mucin binding and prolonged release of the protein antigen, the ALG-coated NPs have the potential to trigger mucosal immune responses. This study highlights the potential use of PLGA NPs in vaccines and the importance of surface functionalization [72]. In another study for polymeric NPs as vaccines, researchers created a vaccine with Chitosan-PLGA NPs loaded with multiepitope peptide, rOmp22, for *Acinetobacter baumannii* infection. The vaccine induced high levels of antibodies and activated immune cells, producing IFN-γ and IL-4, which are essential for immune defense. Compared to non-vaccinated mice, vaccinated mice exhibited lower lung damage and better protection against *A. baumannii*. The vaccine effectively protects against infection by stimulating humoral and cellular immune responses, especially Th1-type responses [73]. In another study on polymeric NPs in vaccines, cationic PLGA NPs were developed and investigated to improve the immunogenicity of foot-and-mouth disease (FMD) DNA vaccines in guinea pigs. Compared to commercial vaccines or bare DNA, NP-delivered DNA vaccines induced higher humoral and cellular immune responses, particularly when coupled with IL-18. The DNA vaccines delivered by NPs provide defense against FMD viral infection, and the most effective formulation was IL-18 [74]. Chitosan NPs, which are polymeric NPs, were investigated as potential carriers in vaccines. AbdelAllah et al. [75] studied alginate-coated chitosan NPs’ potential in the hepatitis A vaccine (HAV). When compared to previous formulations, HAV with alginate-coated chitosan NPs significantly increased splenocyte proliferation, hepatitis A antibodies, and seroconversion rate, along with elevated levels of IFN-γ and IL-10. Furthermore, this formulation was found to be less expensive. The created vaccine increased cellular and humoral immune responses, providing a new potential method for improving HAV vaccinations. For long-term safety and efficacy to be confirmed, more clinical research is needed.

Nanomaterial-based vaccines showed an improvement in combating infectious diseases and new pathogens, from increasing vaccine effectiveness and immunogenicity to allowing targeted delivery and controlled release of antigens. Lipid NPs, polymeric NPs, and other nanostructures are among the nanocarriers that can be used to create vaccines against different diseases. To bring nanomaterial-based vaccines from clinical testing to real-life cases, issues including scalability, stability, safety, and regulatory concerns still need to be addressed.

## 5. Nanomaterials in Immunotherapy

### 5.1. Targeted Drug Delivery Systems for Immunomodulation

Targeted drug delivery refers to delivering drugs or therapeutic agents at a specific site without affecting other cells or tissues, reducing side effects [76]. NPs are very attractive in drug delivery applications since they can improve a drug’s stability, protect it from degradation, increase its time in the circulatory system, and deliver it to the desired location. This increases the drug’s effectiveness, which could lead to an improved treatment [77]. There are a few options for administering the drug by using NPs, all having their advantages and disadvantages, which are presented in Table 3.

Nanocarriers can be produced to encapsulate immunotherapeutic drugs and release them at a specific location within the body. This targeted delivery is achieved through surface modifications with ligands or antibodies that recognize specific biomarkers in immune cells [83]. El-Deeb et al. studied the immunomodulatory effects of polysaccharides-capped silver NPs against *Pseudomonas aeruginosa* [84]. The immunomodulatory effects of silver NPs (AgNPs) were assessed by measuring induced reactive oxygen species (ROS) in ex vivo skin-associated macrophages. Results showed that both *P. aeruginosa* infection and AgNP treatment significantly induced intracellular ROS, with the gating percentage of ROS increasing from 33.46% in untreated animal group cells to 81.27% in the infected-AgNP-treated group. The treatment also disrupted the bacterial outer membrane, reduced biofilm formation, and decreased the production of alginate and pyocyanin. Diez-Echave et al. demonstrated the immunomodulatory properties of silk fibroin NPs loaded with quercetin (QSFN) [85]. QSFN administration significantly downregulated the expression of proinflammatory cytokines in the colonic tissues of treated mice. The reduction in TNF-α, which is a very important mediator of intestinal inflammation, demonstrated QSFN’s potential in modulating inflammatory pathways.

NPs have also been investigated in autoimmune disease treatment. One study focused on the effects of microRNA-125a-loaded polymeric NPs for the treatment of systemic lupus erythematosus (SLE). In SLE patients, miR-125a is significantly reduced in T cells, which leads to an imbalance of T cells. The researchers created a monomethoxy (polyethylene glycol)-poly(D,L-lactide-co-glycolide)-poly(L-lysine) (mPEG-PLGA-PLL) nanodelivery system to deliver miR-125a into splenic T cells. miR-125a-loaded mPEG-PLGA-PLL (PEALmiR-125a) reduced the progression of SLE by restoring the balance between effector T cells and regulatory T cells (Tregs). In contrast, free miR-125a did not have a therapeutic effect. This proved the therapeutic effect of the NP delivery system [86]. In another study, also for an autoimmune disease, vitiligo, the researchers created NPs containing rapamycin and the HEL46-61 peptide (NPHEL46-61/Rapa). The system was tested on mice. They observed that the delivery system promoted the production of Tregs, reduced the levels of the inflammatory cytokines IFN-γ and IL-6, and increased the production of the anti-inflammatory cytokine IL-10. This study provides evidence that NPs carrying rapamycin and autoantigens can be used to induce antigen-specific immune tolerance [87].

### 5.2. Nanoparticles in Cancer Immunotherapy

Since cancer is one of the leading causes of death globally, there is an urgent need for novel therapies. An important effort towards improving cancer drug delivery has been made possible by using nanocarriers [88]. The tumor microenvironment (TME) varies between tumor types, but its main components are specific cells (immune cells, stromal cells, and endothelial cells), extracellular matrix (ECM), and blood vessels. NPs could have the ability to target the major components of TME, by delivering immunostimulatory agents directly to the TME, leading to the activation and proliferation of antitumor immune cells. Additionally, NPs can also be uptaken by APCs, which promote the cytosolic delivery of encapsulated antigens and adjuvants [89,90,91].

**Figure 7:**
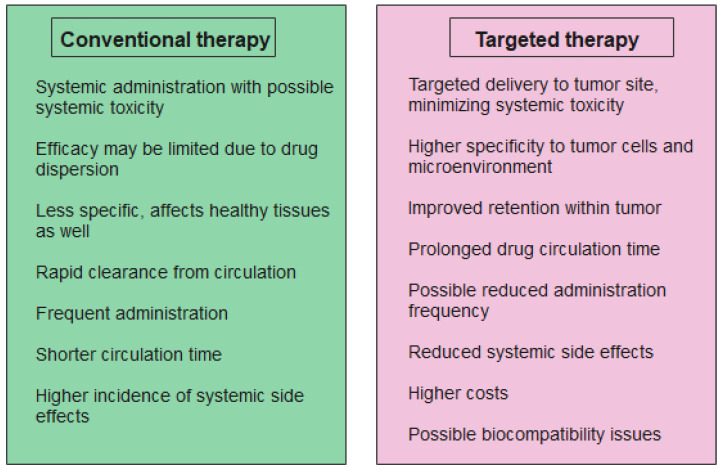
Conventional cancer therapy vs. targeted therapy. Created based on information from [94,95,96].

NPs have several advantages in cancer immunotherapy due to their ability to improve the pharmacokinetics and biodistribution of therapeutic agents. Traditional cancer therapies often have some drawbacks, like limited specificity and off-target effects, which can result in toxicity and reduced efficacy. NPs can deliver the anticancer drugs directly to the tumor site, minimizing systemic exposure and side effects. This ensures that higher concentrations of the therapeutic drug reach the cancer cells and the TME, which could further lead to an improved treatment [91,92,93]. A comparison between targeted cancer therapy and conventional treatment is presented in Figure 7.

One of the strategies in cancer immunotherapy using NPs is the delivery of immune checkpoint inhibitors. These inhibitors, such as anti-programmed cell death protein 1 (anti-PD-1), work by using the body’s immune system to attack cancer cells. However, their systemic administration can lead to immune-related adverse effects. By loading these inhibitors within NPs, researchers could focus their effects on the TME. Studies have shown that NPs can improve the retention and penetration of immune checkpoint inhibitors within tumors [97,98,99]. Zhang et al. investigated the potential of cargo-free PLGA NPs to enhance the efficacy of anti-PD-1 immunotherapy in a mouse model of metastatic triple-negative breast cancer [100]. Their hypothesis was that PLGA NPs, administered intravenously without cargo, could redirect circulating immune cells away from the TME, leading to improved anti-PD-1 therapy. The NPs were found to reduce monocyte chemoattractant protein 1 (MCP-1) expression and increase TNF-α in innate immune cells in vitro. In vivo, they were taken up by myeloid-derived suppressor cells (MDSCs) and monocytes, accumulating primarily in the spleen, liver, lung, and tumor. This changed immune cell distribution reduced MDSC levels in circulation and the lung, slowing tumor growth and improving survival when combined with anti-PD-1 therapy. Gautam et al. studied the potential of cowpea mosaic virus (CPMV) as an in situ vaccination strategy combined with anti-PD-1 peptides for ovarian cancer immunotherapy [101]. It was observed that multivalent display of anti-PD-1 peptides (SNTSESF) combined with CPMV had an improved therapeutic effect compared to free peptides or a combination of soluble peptides with CPMV. This was likely due to the prolonged tumor residence and improved distribution of the NPs compared to the free peptide, which likely undergoes rapid clearance and degradation. Neek et al. created a combination of immune checkpoint inhibition with an E2 NP-based antigen delivery system as an immunotherapy for melanoma [102]. The E2 NP platform was modified to deliver cytosine–guanine (CpG) oligonucleotide 1826 and a glycoprotein 100 (gp100) melanoma antigen epitope (CpG-gp-E2) to increase tumor-specific immune responses. They also combined CpG-gp-E2 with anti-PD-1 therapy. Results demonstrated a high increase in gp100-specific interferon-gamma (IFN-γ), CD8 T cells in the spleen, tumor-infiltrating CD8 T cells, and overall survival compared to monotherapy or untreated controls. Moreover, more than 50% of mice treated with the combination therapy remained cancer-free for a long period of time.

As previously mentioned, NPs can be used to modulate the immunosuppressive components of the TME. Tumors often create an immunosuppressive microenvironment that compromises the effectiveness of immune responses. NPs can deliver agents that reprogram tumor-associated macrophages (TAMs) and MDSCs from a protumorigenic to an antitumorigenic phenotype. In this way, NPs can enhance the infiltration and activity of immune effector cells and improve the overall antitumor immune response [89,103,104]. A recent study explored the use of tumor-associated macrophage membrane-coated NPs (TAMM-NPs) as a novel strategy for cancer immunotherapy. Photodynamic immunotherapy was made possible by coating upconversion NPs with TAMM and conjugating a photosensitizer (NPR@TAMM). The created system was biocompatible, and it effectively switched macrophages from an immunosuppressive M2-like to an inflammatory M1-like phenotype within the TME, inducing immunogenic cell death and enhanced antitumor immunity by stimulating APCs to activate tumor-specific effector T cells. These findings show promise for improving cancer immunotherapies [105]. In another study that focused on targeting the TME, researchers created multifunctional black phosphorus (BP) NPs modified with PEGylated hyaluronic acid (HA) for photo-immunotherapy against malignant tumors. HA-BP NPs demonstrated excellent biocompatibility, stability, and therapeutic efficacy in photothermal therapy (PTT), photodynamic therapy (PDT), and immunotherapy. In vitro experiments showed that HA-BP NPs reduced M2 macrophage marker CD206 expression by 42.3% and increased M1 macrophage marker CD86 by 59.6%, demonstrating their role in converting TAMs to an antitumor phenotype. HA-BPs also accumulate in the tumor through CD44-mediated active targeting and induce immunogenic cell death (ICD). Additionally, it caused the activation of CD4+ and CD8+ T cells in tumors. This NP system demonstrated its efficacy in inhibiting tumor growth and its potential in anticancer therapy [106]. Other studies have highlighted the great effects of NPs in cancer immunotherapy. These studies and their findings are summarized in Table 4.

**Figure 8:**
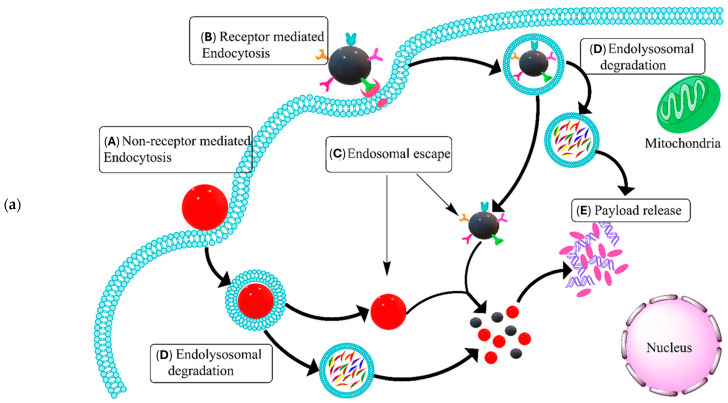

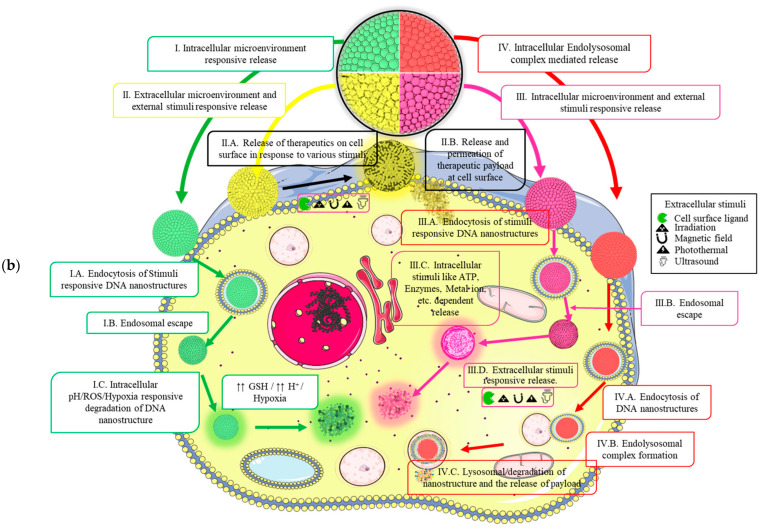
(**a**) Mechanisms of cellular internalization and intracellular delivery of DNA nanostructures: (**A**) non-receptor-mediated endocytosis achieved through caveolin- or clathrin-mediated pathways; (**B**) receptor-mediated endocytosis enabled by surface modification of DNA nanostructures with targeting groups (e.g., peptides, aptamers, antibodies, etc.); (**C**) endosomal escape; (**D**) formation of endolysosomal complex, lysosomal degradation of the DNA-based carrier, and the release of encapsulated therapeutic agents; and (**E**) released therapeutics ready to exert their action in the cellular microenvironment. (**b**) Detailed mechanisms of action of stimuli-responsive DNA nanostructures. Reprinted from an open-access source [115].

Increasing promise in cancer immunotherapy also arises from DNA nanostructures due to their precisely controllable features, remarkable biocompatibility, and simple functionalization [113]. The formation of DNA nanostructures benefits from unprecedented control over structural and functional parameters [114], allowing the targeted delivery of poorly soluble drugs toward maximizing therapeutic efficiency and avoiding cytotoxic effects on normal tissues [115,116]. In general, an electrostatic repulsion between negatively charged DNA and cellular membranes stops DNA from entering the cells. However, DNA nanostructures are easily internalized through various pathways [117], as schematically represented in Figure 8a. In addition, DNA nanostructures can be linked to a broad range of functional moieties that endow them with the ability to selectively release therapeutic cargo in the presence of specific stimuli (Figure 8b). These combinations are extremely valuable in cancer therapy, as DNA nanocarriers can be functionalized to respond to tumor microenvironment cues, leading to enhanced drug concentrations at the targeted site, a faster reach of tumor location, and a lower dosage of therapeutics for achieving the desired effect [115,117]. Specifically, targeted delivery can be refined using ligands (e.g., antibodies, affibodies, aptamers, folates, peptides, and polysaccharides) that enable nanocarriers to recognize and bind to receptors on target cells. Moreover, stimulus-responsive DNA nanostructures can be developed in a manner that responds to numerous external and internal stimuli, including changes in pH, temperature, enzymes, and light. As such, these nanostructures shall undergo some physicochemical transformation within the tumor microenvironment, triggering drug release through conformational changes, disassembly, or degradation. Recent advances in the development of stimuli-responsive DNA sequences, including i-motif and G-quadruplex, have further extended the action of these nanostructures so that the drug release process is spatiotemporally controllable and hence can be performed more effectively for delivering drugs precisely where they are needed, particularly within cancer therapy [115,117,118].

Furthermore, aptamers (single-stranded DNA or RNA molecules containing between 15 and 80 nucleotides) have been explored as potential ligands able to bind a wide range of targets with high affinity and specificity [119,120]. Aptamers offer important advantages over commonly used antibodies, including enhanced thermal stability, lower molecular weight, better tissue penetration, facile synthesis, characterization, and modification, high reproducibility, lack of immunogenicity, and low costs [119,120,121]. Thus, aptamers are considered good candidates for bioanalysis, bioimaging, biosensing, targeted drug delivery, and cancer immunotherapy, providing the necessary means for modulating cellular interactions between immune cells and cancer cells, enhancing antitumor responses, and improving tumor cell recognition [113,119].

Several studies have explored the use of DNA nanostructures as functionalizing agents for various nanoparticles used in cancer immunotherapy. For instance, Saleh et al. [121] have developed an anticancer formulation comprising HER2 aptamer-decorated curcumin-loaded human serum albumin nanoparticles. The proposed nanomedicine has significantly improved the cytotoxicity of CCM in HER2+ cells, offering a promising alternative for treating breast cancer and reducing the side effects of current therapeutic approaches. Yu et al. [122] have proposed a different nanoformulation. The researchers investigated the potential of docetaxel-loaded albumin nanoparticles functionalized with nucleolin-targeted aptamers (AS1411). The described treatment exhibited considerably higher antitumor efficacy and prolonged survival in CT26-bearing model animals compared to non-targeted systems. More recently, Lu et al. [123] have proposed the utilization of an aptamer-driven DNA nanodevice able to augment immunostimulatory activity via facilitation of macrophage uptake and retention of therapeutics. Their study indicated that the analyzed DNA nanodevice reeducates tumor-associated macrophages, leading to the reversal of the tumor immune microenvironment. Therefore, the platform is considered to have high promise for cancer immunotherapy.

These nanoparticle-based immunotherapies hold promise for future new treatments. Many preclinical studies have demonstrated their beneficial effects, such as overcoming immune evasion, increasing therapeutic efficacy, and minimizing systemic toxicity. More research is needed before translation into clinical settings; however, the NP systems provide a great starting point for advancing treatments.

## 6. Safety and Biocompatibility

Biocompatibility is defined as the ability of a material to work in synergy with the body without causing any host responses. In the context of nanomaterials used for immunological applications, the materials should not cause any adverse reactions from the immune system and should provide a therapeutic effect [124,125,126]. However, when nanomaterials cause adverse effects on the immune system, this can be called immunotoxicity. Immunotoxicity can manifest as immunosuppression, where the immune response is weakened, or as immunostimulation, which can lead to excessive immune reactions. Nanomaterials can induce immunotoxicity through certain mechanisms, like the generation of ROS, disruption of cellular membranes, and the activation of inflammatory pathways [126,127]. As mentioned before, the route of exposure can influence the level of toxicity, the biodistribution, and clearance of the NPs. The size, shape, and surface chemistry are other factors that influence the NPs’ toxicity and biocompatibility [128]. In order to create nanomaterials for immunological applications, it is first necessary to understand and overcome their toxic effects. Research has demonstrated the potentially harmful effects that NPs can have in such applications. Table 5 summarizes a few of these findings.

One way to improve NP’s biocompatibility is through surface modification. This can be performed by using specific molecules. One great option is PEG, as it is the most widely used substance for this purpose in biomedical applications [134]. It was approved by the Food and Drug Administration (FDA) for application in the production of medicines and the surface modification of implanted medical devices [135]. A study by Zhen et al. demonstrated the beneficial effect of PEG functionalization of magnetic mesoporous silica NPs (M-MSNs) for delivering CpG-containing oligodeoxynucleotides (ODN) in cancer immunotherapy [136]. The PEG modification reduced the cytotoxicity of the NPs, particularly at concentrations below 100 μg/mL. PEG steric hindrance likely weakened cell–particle interactions, contributing to the reduced toxicity. Additionally, the modified M-MSNs demonstrated excellent immunostimulatory activity in vivo compared to free CpG. Lee et al. synthesized anti-PD-L1 F(ab)-conjugated PEG-PLGA for the delivery of immune checkpoint inhibitors, specifically anti-PD-L1 [137]. The PEG-PLGA NPs had optimal size and surface charge, which are very important factors for cellular uptake and reduced self-aggregation. The system also showed no significant toxicity in vitro and in vivo, which highlights their biocompatibility compared to non-functionalized NPs. The modified size and surface properties of PEG-PLGA NPs reduced renal excretion, increasing their circulation time in the body and leading to an improved therapeutic effect. Another study focused on enhancing the efficacy of T cell-based tumor immunotherapy using dendrimer-entrapped AuNPs (Au DENPs) partially decorated with methoxy polyethylene glycol (mPEG) for the nonviral delivery of CpG oligonucleotides. The biocompatibility of the Au DENPs was significantly improved by PEGylation. According to the study, the PEGylated Au DENPs were less cytotoxic than their non-PEGylated equivalents. Even at high polyplex concentrations, the cell survival of bone marrow-derived dendritic cells (BMDCs) was greater than 75%, demonstrating its biocompatibility [138].

Besides PEG, other existing compounds could improve NP’s biocompatibility and reduce toxicity. One study explored the effect of cell membrane-encapsulated MNPs with a silicon dioxide (SiO_2_) coating on cancer immunotherapy. The SiO_2_ coating improved the colloidal stability and biocompatibility of MNs. By delivering tumor-specific antigens, the cell membrane encapsulation improved natural killer (NK) cell activation and increased the expression of activating receptors. This functionalization increased NK cell antitumor activity while reducing toxicity [139]. In another study, the researchers functionalized an Ag-decorated MoS_2_ nanocomposite with chitosan and investigated it for its antibacterial activity and immunotherapy. Due to its natural biocompatibility, chitosan functionalization provided non-toxic effects and enhanced antibacterial activity. This hybrid nanocomposite demonstrated cytotoxicity against cancer cells with dosage-dependent effects, highlighting its low toxicity [140]. Researchers have also investigated the effect of MSNs loaded with Resiquimod (R848) and biotin-avidin caps to activate local immune responses. This functionalization improved the pharmacokinetic profile of R848, increased half-life, and reduced systemic toxicity. MSNs were efficiently taken up by APCs without showing immunotoxicity up to 100 μg/mL, demonstrating their performance as a biocompatible and effective drug delivery system for immune-activating drugs [141].

Considering all these findings, conducting more research to fully understand nanomaterials’ potentially harmful effects on cells and tissues before they can be used in human medical treatments is essential. However, much progress has been made during the past years, and plenty of information has been gathered about NPs’ modes of action in immunology, which could serve as a base for future innovations.

## 7. Conclusions

Integrating nanomaterials into immunological research significantly improves both fundamental understanding and biomedical applications. This review has covered the roles of nanomaterials in immune modulation, diagnostics, and therapy, demonstrating their versatility across diverse immunological contexts. Nanomaterials, defined as materials with at least one dimension ranging from 1 to 100 nm, have unique properties that can make them useful in different biomedical applications. They can be used in diagnostics since they can improve these processes by increasing accuracy and sensitivity. In this context, NPs can be modified with specific ligands to detect biomarkers or pathogens, resulting in early disease detection and monitoring, even at low concentrations. NPs can also be used in vaccine development, where they can act as adjuvants or as carriers for antigens or nucleic acids. They can protect the loaded agents from degradation and facilitate their delivery, providing a long-lasting immune response against infectious agents.

In therapy, NPs can act as drug delivery systems for immunomodulation, carrying the therapeutic drugs that provide targeted delivery and an increased therapeutic effect. This targeted delivery is achieved through surface modifications with ligands or antibodies that recognize specific biomarkers on immune cells. In addition, nanomaterials can be used in cancer immunotherapy, which is essential since cancer treatment is a very pressing problem worldwide. They have the ability to improve the pharmacokinetics and biodistribution of therapeutic agents. NPs can deliver immunomodulatory agents directly to the TME, where they improve antitumor immune responses and suppress tumor growth. Using NPs in cancer treatment has many advantages compared to traditional treatments, such as higher specificity for tumor cells, retaining the drug at the tumor site for a prolonged period, reducing systemic toxicity, and possibly reducing administration frequency.

Despite nanomaterials’ many advantages and benefits in immunology applications, biocompatibility and safety must first be addressed before their translation into clinical settings. Studies have observed that NPs could have harmful effects on cells, which are usually size- and dose-dependent. Therefore, even more research is needed to address these concerns. Researchers are actively exploring strategies to improve the biocompatibility of nanomaterials through surface modifications, polymer coatings, and the use of biodegradable materials to minimize adverse effects. Long-term safety profiles, scalability of manufacturing processes, and regulatory considerations also need to be addressed to use the full potential of nanomaterials and translate these advancements into clinical settings.

## Figures and Tables

**Table 1:** Research showcasing nanomaterials’ effects in immunodiagnostics.

Nanomaterial	Target	Findings	Ref.
Plasmon color-preserved AuNP clusters	Detection of SARS-CoV-2	Selective detection of the SARS-CoV-2 nucleocapsid protein Detection at low concentrations Improved detection compared with available COVID-19 test kits	[46]
Polypyrrole (nano)structures decorated with AuNPs	COVID-19 serological diagnosis	Provided optimal surfaces for protein attachment Sensitively detected specific antibodies Rapid detection (less than one hour) Low-cost alternative	[47]
Au NPs/MoS_2_-graphene aerogels composite	Detection of prostate-specific antigen	Excellent conductivity, which accelerates the electron transport of the electrode interface Amplified the electrochemical signal Detection at low concentrations Stable and selective	[48]
Au NPs/MWCNTs-graphene quantum dots nanocomposite	Sensitive detection of prostate-specific antigen	Excellent linear relationship between the impedance change and different concentrations of PSA Detection at low concentrations Stable, selective, and reproductible	[49]
Citicoline-bovine serum albumin conjugate and aptamer-functionalized AuNPs nanozyme	Detection of C-reactive protein	High accuracy and sensitivity for detection in blood Low detection limit Low-cost Stability and selectivity	[50]

**Table 2:** Summarization of study findings for LNP-based vaccines.

Targeted Disease	LNPs-Based Vaccine	Findings	Ref.
Influenza	R-DOTAP (1,2-dioleoyl-3-trimethylammonium-propane) cationic LPNs	Outperformed conventional adjuvants in promoting peptide-specific CD4 T cell responses Enhanced immune response through different mechanisms R-DOTAP alone activated and differentiated CD4 T cells	[64]
Influenza A virus of swine (IAV-S)	Hemagglutinin Antigen LPNs	Provided robust systemic and mucosal responses in pigs Induced antibody and T-cell responses led to complete protective immunity in vaccinated pigs	[65]
Lyme disease	mRNA-LNPs	Induced stronger antibody and T-cell responses More robust CD8+ and CD4+ T cell responses Led to higher levels of antigen-specific memory B cells	[66]
SARS-CoV-2 variants	4N4T-LNPs	Showed higher mRNA translation efficiency compared to existing delivery systems Induced stronger immune responses Higher levels of RBD-specific IgG and neutralizing antibodies	[67]
Monkeypox virus (MPXV)	mRNA-LNPs	Induced MPXV-specific antibodies, potent neutralizing antibodies, and efficient No side effects	[68]

**Table 3:** Possible routes of drug administration using NPs. Created based on information from [78,79,80,81,82].

Route of Administration	Advantages	Disadvantages
Oral	Non-invasive High patient compliance	Degradation in the gastrointestinal tract First-pass metabolism Poor absorption
Transdermal	Non-invasive Large surface area for administration Good patient compliance	Possible issues with drug absorption Irritation
Intravenous	Systemic NP delivery Prompt response	Risk of systemic toxicity Pain in the administration area Invasive
Inhalation	Non-invasive Large surface area for administration Avoidance of first-pass metabolism	Possible pulmonary clearance Risk of local toxicity The potential of passing into the systemic circulation
Ophthalmic	Direct delivery to ocular tissues Minimizes systemic side effects	Toxicity in certain conditions Potential irritation Limited to ocular treatments
Intranasal	Non-invasive Direct delivery to the brain	Mucociliary clearance Possible irritation

**Table 4:** Studies for NPs in cancer immunotherapy and their findings.

NP System	Targeted Cancer Type	Findings	Ref.
Resiquimod-loaded platelet membrane-coated nanoparticles	Solid tumors (colorectal and breast cancer models)	Enhanced intratumoral delivery of resiquimod Increase in effector and central memory T cells Significant reduction in lung metastasis	[107]
Biomimetic NPs loaded with mRNAs encoding costimulatory receptors (OX40)	Various tumors	Delivery of OX40 mRNA to T cells enhanced antitumor activity Tumor regression Increased response to checkpoint blockade	[100]
Manganese zinc sulfide NPs	Metastatic melanoma	Induction of ICD Activation of cytotoxic T cells Reprogramming of tumor microenvironment Metastasis inhibition	[108]
Iron oxide nanoparticles (IONPs)	Breast tumors	Local hyperthermia via PTT depletes tumor-associated Tregs Increased efficacy of anti-CTLA-4 immunotherapy Inhibited tumor growth	[109]
Doxorubicin/CTLA-4 blocker-co-loaded liposomes	Various tumors	Promoted CD8+ T cell activation Stimulated cytokine production Effective tumor cell eradication	[110]
Thermosensitive hydrogel (F127-g-gelatin) releasing S-nitroso glutathione (GSNO) and anti-CTLA-4 micelles	Melanoma	Sustained release of GSNO and anti-CTLA-4 within the tumor microenvironment Potentiation of abscopal effects Improved antitumor immune response	[111]
Selenium-containing NPs with doxorubicin	Lung metastasis and subcutaneous tumors	Radiation-responsive SeNPs release selenic acid Increased NK cell-mediated immunotherapy Targeted doxorubicin delivery	[112]

**Table 5:** Study findings for the toxicity of NP systems.

NP System	Toxic Effect	Ref.
Chitosan-coated AgNPs	Dose-dependent toxicity in rats, severe at 50 mg/kg with hepatic and renal congestion Oxidative stress is evidenced by increased malondialdehyde (MDA) and decreased glutathione (GSH) Elevated liver enzymes (ALT, AST) and altered protein levels Renal damage is indicated by increased urea nitrogen and creatinine levels	[129]
Copper oxide NPs (CuO NPs)	Toxic effects on zebrafish embryos Decreased heartbeat Gene expression changes related to antioxidant and immune systems Increased mortality	[130]
Copper nanoparticles (Cu NPs)	Inhibition of growth in Takifugu fasciatus Increased oxidative stress Altered mitochondrial function Immune response activation	[131]
Zinc oxide (ZnO) NPs	Size- and dose-dependent cytotoxicity in HepG2 cells Release of Zn^2+^ Increased oxidative stress Inflammatory response	[132]
Different NPs (nZnO, nFe_2_O_3_, nCuO, and MWCNT)	ROS induction DNA damage Alteration of neurotransmitter levels and gene expressions related to immune and neurotransmitter systems	[133]

## Data Availability

Not applicable.
